# Biology and Clinical Implications of Fecal Occult Blood Test Screen-Detected Colorectal Cancer

**DOI:** 10.1093/jncics/pkab100

**Published:** 2022-01-10

**Authors:** Shehara Mendis, Wei Hong, Sumitra Ananda, Ian Faragher, Ian Jones, Matthew Croxford, Malcolm Steel, Azim Jalali, Grace Gard, Yat Hang To, Margaret Lee, Suzanne Kosmider, Rachel Wong, Jeanne Tie, Peter Gibbs

**Affiliations:** 1 Personalised Oncology Division, Walter & Eliza Hall Institute, Parkville, VIC, Australia; 2 Department of Medical Oncology, Sunshine Hospital, Western Health, St Albans, VIC, Australia; 3 Faculty of Medicine & Health Sciences, The University of Melbourne, Parkville, VIC, Australia; 4 Department of Medical Oncology, Peter MacCallum Cancer Centre, Parkville, VIC, Australia; 5 Department of Colorectal Surgery, Footscray Hospital, Western Health, Footscray, VIC, Australia; 6 Department of Colorectal Surgery, The Royal Melbourne Hospital, Parkville, VIC, Australia; 7 Department of Surgery, The University of Melbourne, Parkville, VIC, Australia; 8 Department of Colorectal Surgery, Box Hill Hospital, Eastern Health, Box Hill, VIC, Australia; 9 Department of Medical Oncology, Eastern Health, Box Hill, VIC, Australia; 10 Eastern Health Clinical School, Monash University, Box Hill, VIC, Australia; 11 Department of Medical Oncology, The Royal Melbourne Hospital, Parkville, VIC, Australia

## Abstract

**Background:**

Fecal occult blood test (FOBT)–based screening for colorectal cancer (CRC) reduces mortality, with earlier stage at diagnosis a prominent feature. Other characteristics of FOBT screen-detected cancers and any implications for clinical management have not been well explored.

**Methods:**

We examined a multisite clinical registry to compare the characteristics and outcomes of FOBT screen-detected CRC via the Australian National Bowel Cancer Screening Program (NBCSP), which is offered biennially to individuals aged 50-74 years, and age-matched non-screen-detected CRC in the same registry. All statistical tests were 2-sided. Odds ratios (ORs) were calculated using the Baptista-Pike method, and hazard ratios via the log-rank method.

**Results:**

Of 7153 registry patients diagnosed June 1, 2006, to June 30, 2020, 4142 (57.9%) were aged between 50 and 74 years. Excluding 406 patients with non-NBCSP screen-detected cancers and 35 patients with unknown method of detection, 473 (12.8%) were screen detected via the NBCSP, and 3228 (87.2%) were non-screen detected. Screen-detected patients were younger (mean age = 62.4 vs 64.2 years; *P* < .001) and more medically fit (OR for ASA score 1-2 = 1.91, 95% confidence interval [CI] = 1.51 to 2.41; *P* < .001). Pathologic characteristics within each stage favored the screen-detected patients. Stage III screen-detected colon cancers were more likely to receive adjuvant therapy (OR = 3.58, 95% CI = 1.52 to 8.36; *P* = .002). Screen-detected patients had superior relapse-free (hazard ratio = 0.41, 95% CI = 0.29 to 0.60; *P* < .001) and overall survival (hazard ratio = 0.22, 95% CI = 0.15 to 0.35; *P* < .001), which was maintained in matched stage comparisons and multivariable analysis.

**Conclusions:**

Beyond stage at diagnosis, multiple other factors associated with a favorable outcome are observed in FOBT screen-detected CRC. Given the substantial stage-by-stage differences in survival outcomes, if independently confirmed, individualized adjuvant therapy and surveillance strategies could be warranted for FOBT screen-detected cancers.

Colorectal cancer (CRC) is the third most commonly diagnosed cancer worldwide and the second most common cause of cancer death (1). CRC survival is stage dependent (2). The earliest stage of CRC is managed with surgery alone, whereas more advanced stages require multimodality approaches. In the metastatic setting, 5-year survival rates are poor at around 13% (2,3).

Fecal occult blood testing (FOBT) provides a noninvasive screening method for presymptomatic CRC diagnosis through the detection of clinically occult bleeding. Improved overall survival (OS) has consistently been demonstrated in patients with FOBT screen-detected cancers when compared with their non-screen-detected counterparts, in both randomized controlled trials and large cohort studies (4-10). A stage migration effect, whereby more early stage lesions are detected via screening, is well documented (11,12) and considered the major determinant of survival gains.

Australia’s National Bowel Cancer Screening Program (NBCSP) uses an automatic mail out of FOBT test kits, which are now sent to all individuals aged 50 to 74 years on a biennial basis (13). Here, we examine the clinicopathologic characteristics of screen-detected vs non-screen-detected CRC, exploring beyond stage at diagnosis (10,14) and considering any impact on survival outcome and any potential implications for clinical management of the patient with FOBT screen-detected CRC.

## Methods

### Study Design

We assessed an Australian cohort of NBCSP screen-eligible patients with CRC (aged 50-74 years at diagnosis) whose cancers were either screen detected through the NBCSP or non-screen detected, with respect to overall and stage-specific survival outcomes. Secondary questions included exploring differences in patient, tumor, or treatment characteristics that could be contributory to any observed differences in survival outcomes. We hypothesized that differences beyond stage at diagnosis may be contributing to superior survival outcomes. If observed, this understanding may potentially have clinical implications for the patients diagnosed via FOBT-based screening.

### Patients

This study used the Australian Comprehensive Cancer Outcomes and Research Database-Colorectal Cancer (ACCORD CRC), an electronic multisite registry that has prospectively captured data on consecutive colorectal cancer patients at public and private hospitals in metropolitan Melbourne since 2003 (15). Data are held at participating sites and centrally linked in a de-identified manner for research purposes using BioGrid Australia software (16). This registry has ethics committee approval through the Melbourne Health Human Research Ethics Committee, and each individual project undergoes a separate ethics application process through BioGrid Australia (Project ID 202006/4).

Data from 3 public hospital and 3 private hospital ACCORD-CRC databases were used. Patients aged between 50 and 74 years and diagnosed between June 1, 2006, and June 30, 2020, were included to match the targeted demographic of the NBCSP. CRC that were detected via an alternate screening program that was outside the NBCSP, such as endoscopic screening for a family history of CRC, were initially excluded from primary analyses. These non-NBCSP screen-detected patients were subsequently included as a separate cohort in survival and multivariable analyses. Patients with multiple primary tumors (synchronous or metachronous) were also excluded.

### Variables

Demographics collected were age at initial diagnosis, sex, and Index of Relative Socioeconomic Advantage and Disadvantage (IRSAD) as determined by the patient’s residential postcode, with IRSAD 1 indicating most disadvantage and IRSAD 10 indicating the most advantage. The American Society of Anesthesiologists physical status classification (ASA score) and Eastern Cooperative Oncology Group Performance Status (ECOG PS) were used to assess overall medical fitness. Primary tumor site (right colon, left colon, rectum), tumor (T) stage, and nodal (N) stage as per the American Joint Committee on Cancer (AJCC) staging system were collected.

Poor prognostic features assessed included poor tumor differentiation and lymphovascular invasion for all tumor stages. Additional high-risk features for AJCC stage II colon cancers were at least 1 of the following: emergency presentation, T4, lymphovascular invasion or fewer than 12 lymph nodes examined (17,18). High-risk features for AJCC stage III colon cancers were T4 and/or N2 disease (19). Receipt of chemotherapy and resection of primary and any metastatic disease (at diagnosis or after later relapse) were analyzed.

### Assessments

Clinical outcomes collected were relapse-free survival (RFS), OS, and CRC-specific mortality. RFS in stages I-III was the period from date of diagnosis to date of first relapse or death in the absence of known relapse. OS was defined as the time from date of first diagnosis of CRC to date of death, with censoring at time of last follow-up if no date of death was recorded. Deaths were recorded as due to CRC vs other causes.

### Statistical Analysis

Comparison of the tumor and treatment characteristics of the NBCSP screen-detected group and the control group of non-screen detected patients was performed using unpaired *t* tests with Welch correction for numerical data and χ^2^ tests for categorical data. Fisher exact test was used when counts were below 10. Odds ratios (ORs) and 95% confidence intervals (CIs) were used to determine effect size using the Baptista-Pike method. Three-way logistic regression was used to compare primary tumor side between groups.

Median survival comparisons were carried out using Kaplan-Meier survival estimates. For RFS and OS, hazard ratios (HRs) were calculated using the log-rank method. Univariate analysis was performed to identify clinicopathologic variables associated with OS. For CRC-specific OS, subhazard ratios were calculated using competing risks regression according to the Fine-Gray proportional hazards model (20), adjusted for age, sex, ECOG PS, primary tumor location, stage, grade, mucinous differentiation, lymphovascular invasion, and diagnosis date. Kaplan-Meier survival estimates were used to verify the proportional hazards assumption. Two-sided *P* values less than .05 were considered statistically significant. Data analysis was primarily carried out using GraphPad Prism, v8.2.1 (GraphPad, La Jolla, CA), with Stata Version 15.1 (Stata Corp, College Station, TX) used for regression analyses.

## Results

### Study Population

**Figure 1.**
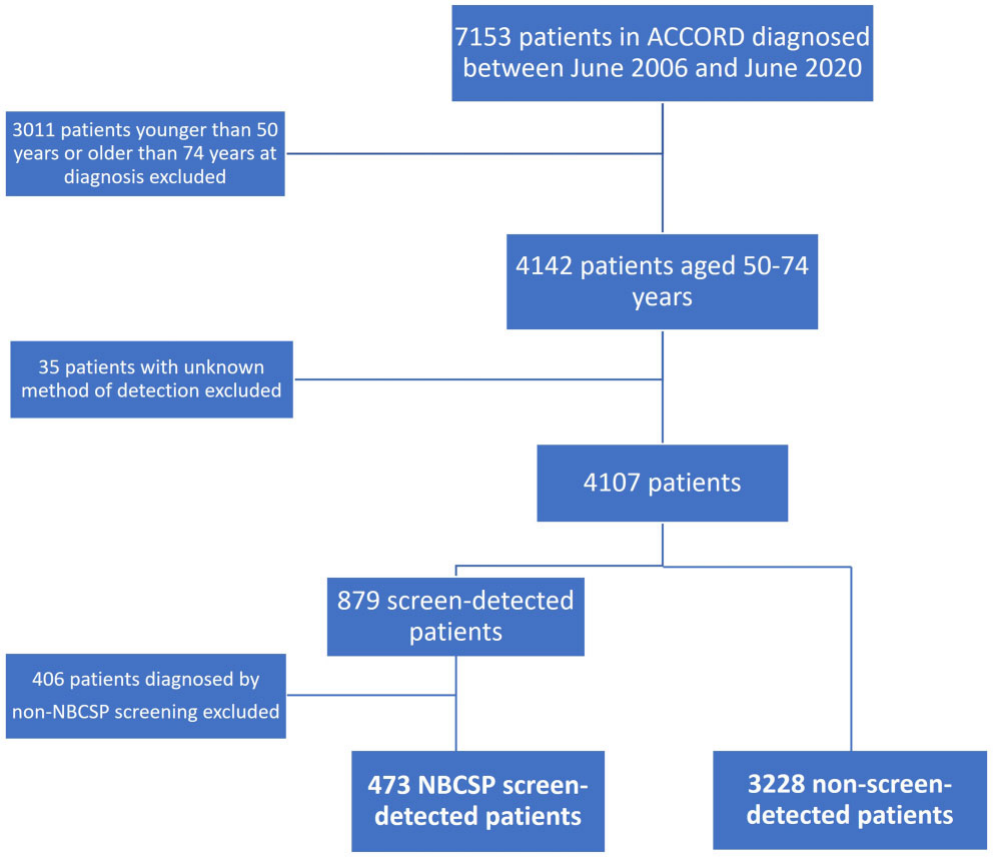
Consort diagram. ACCORD = Australian Comprehensive Cancer Outcomes and Research Database-Colorectal Cancer; NBCSP = National Bowel Cancer Screening Program.

There were 7153 patients within the ACCORD database diagnosed with CRC between June 1, 2006, and June 30, 2020 (Figure 1). Of these patients, 4142 (57.9%) were aged between 50 and 74 years. Of these, we excluded 35 patients where method of CRC detection was not recorded and 406 patients who had cancers diagnosed via screening methods outside of the NBCSP, leaving 473 (12.8%) NBCSP screen-detected patients and 3228 (87.2%) non-screen-detected patients. Of the 3228 non-screen-detected patients, 3160 (97.9%) presented symptomatically and 68 (2.1%) were incidental findings on investigation of an unrelated condition.

### Patient Demographics and Characteristics

Patient demographics and characteristics are shown in Table 1. Screen-detected patients were younger than non-screen-detected patients (mean age = 62.4 vs 64.2 years; *P* < .001). There was no difference in sex distribution or IRSAD ranking between the screen-detected and non-screen-detected groups. Screen-detected patients had a more favorable ASA score than non-screen-detected patients, with ASA score 1-2 in 78.9% of screen-detected vs 66.0% of non-screen-detected patients (OR = 1.91, 95% CI = 1.51 to 2.41; *P* < .001), and included more patients with an ECOG PS of 0-1 (94.5% vs 86.4%, OR = 4.51, 95% CI = 2.30 to 9.42; *P* < .001). Median follow-up was 30 (interquartile range = 10-59) months for screen-detected patients and 52  (interquartile range = 24-72) months for non-screen-detected patients.

**Table 1.** Demographics, patient, and tumor characteristics of patients with National Bowel Cancer Screening Program screen-detected and non-screen-detected colorectal cancer

Characteristic	Screen-detected CRC	Non-screen-detected CRC	*P*
Total, No. (%)	473 (12.8)	3228 (87.2)	
Mean age at diagnosis (min, max)	62.4 (50.2, 74.8)	64.2 (50.0, 75.0)	
Age at diagnosis, No. (%), y			<.001^a^
50-59	167 (35.3)	892 (27.6)	
60-69	218 (46.1)	1530 (47.4)	
≥70	88 (18.6)	805 (25.0)	
Sex			
Female	195 (41.2)	1305 (40.4)	.75^b^
Male	278 (58.8)	1922 (59.6)	
IRSAD, No. (%)			.93^b^
1-4	87 (18.4)	585 (18.1)	
5-7	169 (35.7)	1196 (37.1)	
8-10	205 (43.3)	1417 (43.9)	
ASA score, No. (%)^c^			<.001^b^
1-2	373 (78.9)	2129 (66.2)	
3-5	100 (21.1)	1089 (33.7)	
ECOG PS			<.001^b^
0-1	447 (94.5)	2790 (86.5)	
≥2	8 (1.7)	225 (7.0)	
Unknown	18 (3.8)	212 (6.6)	—
Primary tumor site			<.001^d^
Right colon	147 (31.1)	992 (30.7)	
Left colon	194 (41.0)	1050 (32.5)	
Rectum	132 (27.9)	1149 (35.6)	
Tumor differentiation			
Well-moderately differentiated	361 (76.3)	2144 (66.4)	<.001^b^
Poor-undifferentiated	60 (12.7)	588 (18.2)	
Unknown	52 (11.0)	495 (15.3)	—
Mucinous differentiation			
Yes	76 (16.1)	636 (19.7)	.002^b^
No	315 (66.6)	1748 (54.2)	
Unknown	82 (17.3)	843 (26.1)	—
Lymphovascular invasion			
Yes	121 (25.6)	875 (27.1)	.24^b^
No	331 (70.0)	2095 (64.9)	
Unknown	21 (4.4)	257 (8.0)	—
T stage			<.001^b^^,d^
T0-T1	162 (34.2)	398 (12.3)	
T2	78 (16.5)	396 (12.3)	
T3	166 (35.1)	1501 (46.5)	
T4	42 (8.9)	546 (16.9)	
Tx (unknown)	25 (5.3)	386 (12.0)	—
N stage			.02^b^^,d^
N0	278 (58.8)	1636 (50.7)	
N1	107 (22.6)	732 (22.7)	
N2	46 (9.7)	431 (13.4)	
Nx (unknown)	42 (8.9)	428 (13.3)	—
AJCC stage^e^			<.001^b^
I	182 (38.5)	562 (17.4)	
II	100 (21.1)	921 (28.5)	
III	134 (28.3)	854 (26.5)	
IV	26 (5.5)	666 (20.6)	

a Unpaired *t* test with Welch correction with 2-sided *P* value. AJCC = American Joint Committee on Cancer; ASA = American Society of Anesthesiologists; CRC = colorectal cancer; ECOG PS = Eastern Cooperative Oncology Group Performance Status; IRSAD = Index of Relative Socio-economic Advantage and Disadvantage.

b χ^2^ tests were used for categorical data, unless counts were below 10, in which case Fisher exact test was used. *P* values were 2-sided. Patients with missing data for that category were excluded from analysis.

c ASA physical status classification.

d Multiple logistic regression with 2-sided *P* values were used.

e Note that pathologic staging and detailed histopathologic examination of the primary tumor and locoregional nodes do not routinely occur in de novo stage IV disease.

### Tumor Characteristics

Screen-detected CRC was most often located in the left colon (Table 1), whereas the highest proportion of non-screen-detected CRC was located in the rectum (*P* = .001). Compared with non-screen-detected cancers, screen-detected CRC was more often reported to be well or moderately differentiated (76.3% vs 66.4%, OR = 1.65, 95% CI = 1.24 to 2.19; *P* < .001) and less frequently had mucinous differentiation noted (16.1% vs 19.7%, OR = 0.66, 95% CI = 0.51 to 0.87; *P* = .002). Reported rates of lymphovascular invasion did not differ for screen-detected vs non-screen-detected patients (25.6% vs 27.1%; *P* = .24).

Stage I screen-detected CRC was more often T1 compared with stage I non-screen-detected CRC (66.5% vs 43.4%, OR = 2.78, 95% CI = 1.93 to 4.00; *P* < .001). Stage II screen-detected CRC was more often T3 compared with stage II non-screen-detected CRC (92.0% vs 84.7%, OR = 2.31, 95% CI = 1.06 to 5.15; *P* = .03). Stage II screen-detected colon cancers had a similar rate of high-risk features as stage II non-screen-detected colon cancers (40.5% vs 51.1%; *P* = .09). Stage III screen-detected colon cancers were more often T1 or T2 rather than T3 or T4 compared with stage III non-screen-detected colon cancers (35.6% vs 13.4%, OR = 3.56, 95% CI = 2.39 to 5.27; *P* < .001). Stage III screen-detected colon cancers also had lower rates of N2 disease (19.1% vs 31.1%, OR = 0.52, 95% CI = 0.33 to 0.84; *P* = .007) and were less likely to have other high-risk features than stage III non-screen-detected colon cancers (36.9% vs 50.8%, OR = 0.57, 95% CI = 0.37 to 0.87; *P* = .009).

There was more early-stage CRC in the screen-detected group (AJCC I, II, III, IV = 38.5%, 21.1%, 28.3%, 5.5% vs 17.4%, 28.5%, 26.5%, 20.6%, respectively; *P* < .001) (Table 1). This difference remained statistically significant irrespective of rectal cancer inclusion in the analysis (where a proportion of patients were designated as locally advanced based on initial magnetic resonance imaging). Mismatch repair status was unknown in 89.2% of all patients, therefore comparisons were not performed.

### Treatment Characteristics

The primary tumor was resected more frequently in the screen-detected group compared with the non-screen-detected group (93.9% vs 88.4%, OR = 2.01, 95% CI = 1.36 to 2.99; *P* < .001). Patients who relapsed or were de novo stage IV more often underwent metastatic resection if their primary cancer was initially screen detected (46.2% vs 25.1%, OR = 2.57, 95% CI = 1.46 to 4.45; *P* < .001) compared with non-screen detected. Adjuvant chemotherapy use was not statistically significantly different between stage II screen-detected and non-screen-detected colon cancer patients (14.9% vs 20.9%; *P* = .17). Stage III screen-detected colon cancer patients more often received adjuvant chemotherapy (95.7% vs 87.3%, OR = 3.58, 95% CI = 1.52 to 8.36; *P* = .002) and more often received oxaliplatin-based chemotherapy (82.9% vs 71.7%, OR = 2.27, 95% CI = 1.34 to 3.92, *P* = .002) than non-screen-detected stage III colon cancer patients.

### Survival Outcomes

**Figure 2.**
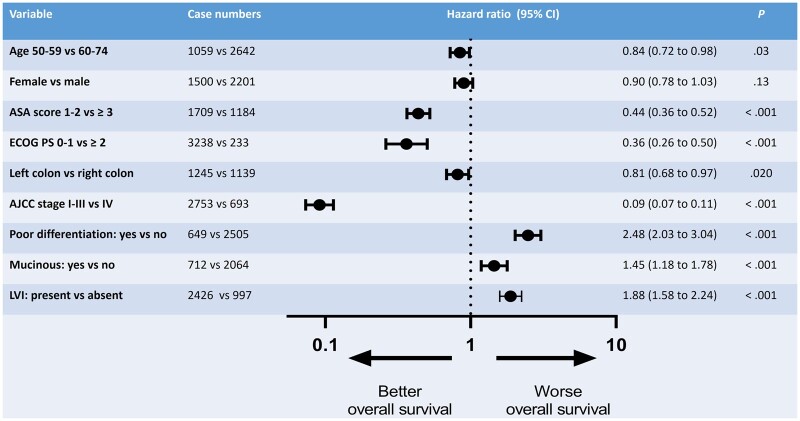
Forest plot for factors associated with overall survival. Hazard ratios and 2-sided *P* values were calculated using the log-rank method. The error bars indicate the 95% confidence intervals (CI). AJCC = American Joint Committee on Cancer; ASA score = American Society of Anesthesiologists physical status classification; ECOG PS = Eastern Cooperative Oncology Group Performance Status; LVI = lymphovascular invasion.

OS was better in younger and fitter patients, those with left-sided disease and early-stage disease, and those without poor differentiation, mucinous histology, or lymphovascular invasion present in their histological specimen (Figure 2).

**Figure 3.**
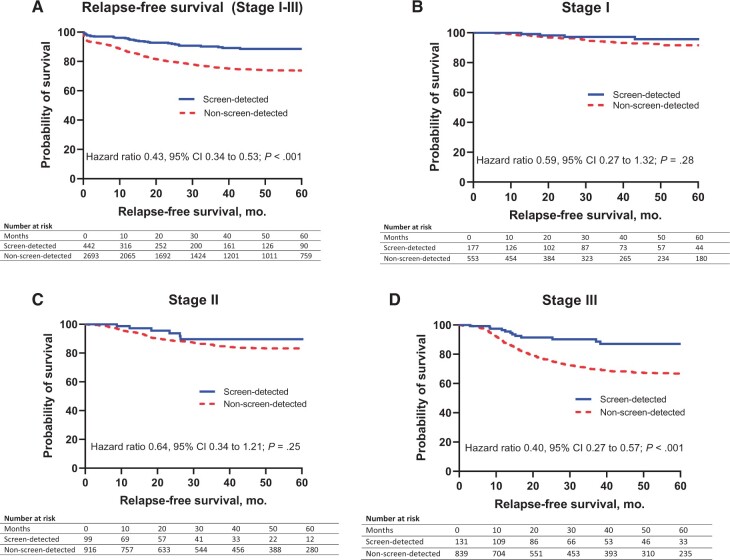
Relapse-free survival. **(A)** Kaplan-Meier survival estimates for 5-year relapse-free survival (RFS) for stages I-III National Bowel Cancer Screening Program (NBCSP) screen-detected colorectal cancers (CRC) vs stages I-III non-screen-detected CRC. **(B, C, D)** 5-year RFS for stages I, II, and III NBCSP screen-detected vs non-screen-detected CRC, respectively. Hazard ratios and 2-sided *P* values were calculated using the log-rank method. CI = confidence interval.

**Figure 4.**
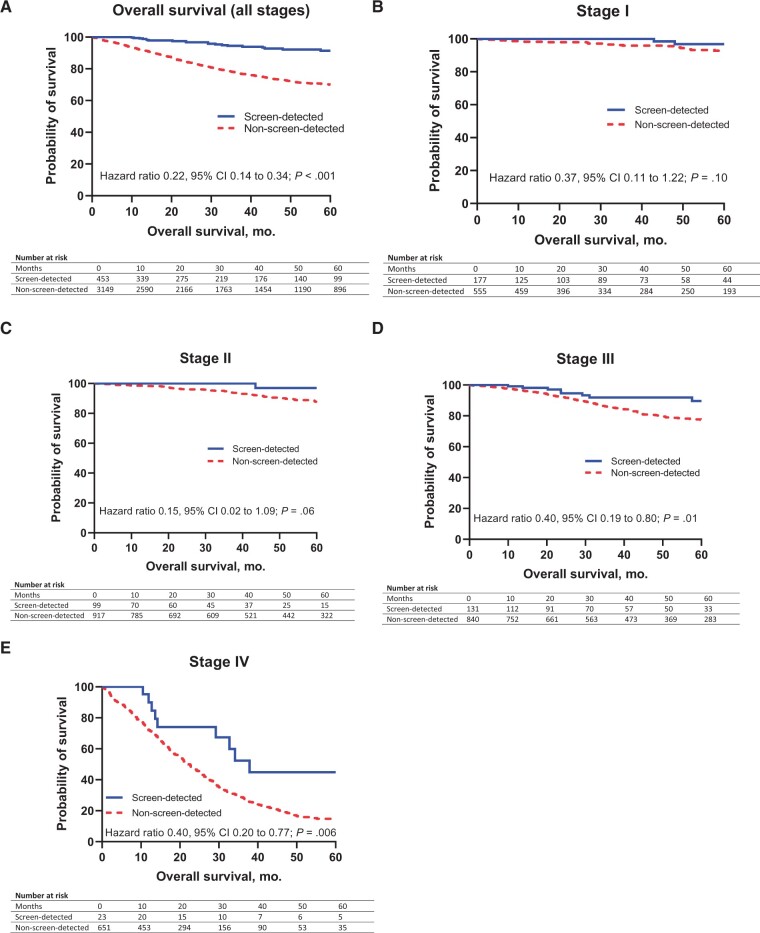
Overall survival. **(A)** Kaplan-Meier survival estimates for 5-year overall survival (5-year OS) for all National Bowel Cancer Screening Program (NBCSP) screen-detected colorectal cancers (CRC) were 91% vs 70% for all non-screen-detected CRC. Median survival was undefined in both groups. **(B, C, D, E)** Five-year OS for stages I, II, III, and IV NBCSP screen-detected vs non-screen-detected CRC, respectively. Median survival was 38 vs 22 months for stage IV screen-detected compared with non-screen-detected CRC. Hazard ratios and 2-sided *P* values were calculated using the log-rank method. CI = confidence interval.

The log-rank hazard ratio for RFS was lower for screen-detected patients compared with non-screen-detected patients (HR = 0.43, 95% CI = 0.34 to 0.53; *P* < .001) ( Figure 3, A). In stage-specific analyses (Figure 3, B-D*)*, the hazard ratio for relapse favored screen-detected over non-screen-detected CRC at each stage, with statistical significance seen in stage III (HR = 0.40, 95% CI = 0.27 to 0.57; *P* < .001; Figure 3, D). OS was superior for screen-detected compared with non-screen-detected CRC (HR = 0.22, 95% CI = 0.14 to 0.34; *P* < .001), with a 5-year OS of 91% for screen-detected and 70% for non-screen-detected patients (Figure 4, A). OS continued to favor screen-detected patients over non-screen-detected patients in matched stage comparisons (Figure 4, B-E) with statistical significance demonstrated in stages III (HR = 0.39, 95% CI = 0.19 to 0.80; *P* = .01; Figure 4, D) and IV (HR = 0.39, 95% CI = 0.20 to 0.77; *P* = .006; Figure 4, E). Five-year OS for stages I and II screen-detected CRC was the same (97% for both). Stage II screen-detected CRC had better 5-year OS rates than stage I non-screen-detected CRC (97% vs 92%). Stage III screen-detected patients had similar 5-year OS to stage II non-screen-detected (90% vs 88%) patients.

Among the subset of high-risk stage II colon cancers, RFS and OS numerically favored screen-detected patients over non-screen-detected patients (HR = 0.23, 95% CI = 0.083 to 0.63; *P* = .11, for RFS; HR = 0.34, 95% CI = 0.10 to 1.2; *P* = .09, for OS; data not shown). For high-risk stage III colon cancers, RFS and OS also numerically favored screen-detected patients over non-screen-detected patients (HR = 0.51, 95% CI = 0.29 to 0.91; *P* = .08, for RFS; HR = 0.39, 95% CI = 0.18 to 0.83; *P* = .09, for OS; data not shown).

Screen-detected colon cancers and screen-detected rectal cancers had superior OS to their non-screen-detected counterparts (HR = 0.22, 95% CI = 0.17 to 0.29; *P* < .001, for colon cancers; HR = 0.22, 95% CI = 0.14 to 0.35; *P* < .001, for rectal cancers; data not shown).

### Screen-Detected CRC Outside the NBCSP

There were 406 patients that were screen-detected outside the NBCSP in the database; 185 (46%) were detected via FOBT, and 221 (54%) were detected endoscopically. This population as slightly older and less fit as measured by ASA score compared with the NBCSP screen-detected population and were more likely to have right-sided tumors (Supplementary Table 1, available online). Other demographics and tumor characteristics for non-NBCSP screen-detected CRC were not statistically different to NBCSP screen-detected CRC. Kaplan-Meier survival estimates numerically suggested a trend toward RFS and OS of the non-NBCSP screen-detected patients being superior to non-screen-detected patients but inferior to NBCSP screen-detected patients (Supplementary Figures 1 and 2, available online*)*.

### Multivariable Analysis of OS

A multivariable analysis using a competing risks model for CRC-specific survival was performed that included the NBCSP screen-detected, non-NBCSP screen-detected, and non-screen-detected groups. The model initially adjusted for all clinicopathologic factors associated with OS: age, sex, ASA, ECOG PS, primary tumor location, stage at diagnosis, tumor grade, mucinous differentiation, and lymphovascular invasion. Stage II patients were broken down into T3 and T4 subgroups, and stage III patients were broken down into low- and high-risk subgroups. There was a proportional hazards violation when ASA and stage IV patients were included; therefore the model was changed to exclude ASA and stage IV patients. To control for population drift, patients were also divided into those diagnosed in the first, second, and third parts of the study period.

Altogether there were 541 cancer deaths. This fact showed that OS still strongly favored NBCSP screen-detected compared with non-screen-detected CRC, with a hazard ratio for CRC-specific death of 0.38 (95% CI = 0.16 to 0.86; *P* = .02) for screen-detected patients (Table 2). In this multivariable model, the hazard ratio for death for non-NBCSP screen-detected CRC was 1.12 (95% CI = 0.68 to 1.85; *P* = .67), indicating no statistically significant difference in survival on multivariable analysis between the non-NBCSP screen-detected group and the non-screen-detected group.

**Table 2.** Multivariable analysis for colorectal cancer (CRC)–specific survival for stage I-III patients

Variable	Sub-HR for CRC-specific survival (95% CI)	*P* ^a^
Method of detection		
Non-screen detected	1	
Screen detected via NBCSP	0.38 (0.16 to 0.86)	.02
Screen detected outside of the NBCSP	1.12 (0.68 to 1.85)	.66
Age, y		
50-59	1	
60-69	1.01 (0.71 to 1.43)	.96
70-74	1.17 (0.78 to 1.76)	.45
Sex		
Female	1	
Male	0.95 (0.71 to 1.27)	.72
ECOG PS		
0-1	1	
≥2	2.14 (1.24 to 3.68)	<.001
Primary tumor location		
Left colon	1	
Right colon	1.27 (0.89 to 1.82)	.18
Rectum	1.40 (0.96 to 2.04)	.08
AJCC stage		
I	1	
IIA (T3N0)	1.96 (1.07 to 3.59)	.03
IIB-C (T4N0)	6.12 (3.06 to 12.26)	<.001
III, low risk (T1-3, N1)	2.85 (1.57 to 5.19)	.001
III, high risk (T4 and/or N2)	8.35 (4.68 to 14.89)	<.001
Tumor grade		
Well-moderately differentiated	1	
Poor-undifferentiated	1.41 (1.01 to 1.97)	.04
Unknown	1.38 (0.67 to 2.86)	.38
Mucinous differentiation		
No	1	
Yes	1.30 (0.91 to 1.86)	.16
Unknown	1.15 (0.80 to 1.64)	.46
Lymphovascular invasion		
No	1	
Yes	1.19 (0.85 to 1.66)	.30
Unknown	1.81 (0.71 to 4.64)	.21
Diagnosis period		
2006-2010	1	
2011-2015	1.04 (0.75 to 1.44)	.81
2016 onward	0.66 (0.36 to 1.19)	.17

a Fine-Gray competing risks analysis for CRC-specific survival used with 2-sided *P* values. AJCC = American Joint Committee on Cancer; CI = confidence interval; ECOG PS = Eastern Cooperative Oncology Group Performance Status; NBCSP = National Bowel Cancer Screening Program; sub-HR = subhazard ratio.

## Discussion

To our knowledge, this is the first study using prospectively collected multi-institutional data to provide a comprehensive assessment of patient, tumor, treatment, and outcome data for CRC detected by FOBT within a national screening program vs patients detected outside of a screening program. Along with the previously widely reported difference in stage at diagnosis for screen-detected vs non-screen-detected patients, we found multiple other differences. Clinical and tumor characteristics and clinical management differences consistently favored screen-detected vs non-screen-detected patients. RFS and OS of patients with screen-detected CRC were more favorable than those with non-screen-detected CRC in all stage-for-stage comparisons, because of improved CRC-specific mortality. This survival benefit was independent of primary tumor site.

We observed differences in the patient characteristics of NBCSP screen-detected vs non-screen-detected patients, with the former being statistically significantly younger and fitter, as measured by the ASA score and the ECOG PS (Figure 2). These factors were associated with superior OS outcomes in the overall patient population. In multivariable analysis adjusting for clinicopathologic factors associated with improved survival, as well as adjusting for within-stage migration, NBCSP screen-detected cancers still had statistically significantly improved CRC-specific survival compared with non-screen-detected cancers. Patients with cancers that were screen-detected outside of the NBCSP did not have improved survival compared with non-screen-detected patients on multivariable analysis, once patient and tumor factors were accounted for. Therefore, survival benefits in this study were particularly seen in the cohort that engaged in a population-level screening program.

Along with more early-stage cancers diagnosed in screen-detected patients, we consistently observed better prognostic features within each stage, consistent with a concept of within-stage progression over time, prior to progression to a higher stage. Stages I and II screen-detected tumors had earlier T stages than their non-screen-detected counterparts, and stage III colon cancer screen-detected patients were more likely to have an earlier T stage or N stage than non-screen-detected patients.

Beyond stage at diagnosis, screen-detected tumors also carried more favorable histological features such as a moderate-to-high degree of differentiation and less mucinous differentiation (21). Although it has been previously suggested, what we could not explore with our data was whether screen-detected cancers are likely to be more slow growing than non-screen-detected ones, thereby resulting in length time bias also contributing to improved survival outcomes (22,23).

Multidisciplinary management also varied when comparing screen-detected and non-screen-detected patients. Patients with stage III screen-detected colon cancers were more likely to receive adjuvant chemotherapy, which improves survival (24–27). For de novo stage IV patients, the screen-detected group was more likely to have primary tumor and distant oligometastatic disease resected, interventions driven by curative intent management. Overall these differences in multidisciplinary care are also likely to be contributing to the superior survival of stages III and IV screen-detected patients.

The hazard ratios for relapse in stages I, II, and III were numerically superior for screen-detected patients, though statistical significance was not reached in stages I or II, likely because of the limited number of events in this group and the limited sample size (type II statistical error). Similarly for OS, statistically significant differences were seen only for stages III and IV.

The stage-by-stage comparisons are most striking when comparing data across stages (Figures 3 and 4). Stage II screen-detected patients had numerically better 5-year OS rates (97%) than non-screen-detected stage I patients (92%), suggesting that screen-detection could be a marker of stage II patients who should not be offered adjuvant chemotherapy because of the already excellent prognosis. Notably, the 5-year OS survival in these patients is superior in our series to other reports of outcomes for deficient mismatch repair patients, which is an accepted marker of stage II patients who should not be considered for adjuvant treatment, although in part, this reflects uncertainty as to the benefit of 5-fluorouracil–based chemotherapy in this molecular subtype (28).

Stage III screen-detected CRC patients had a comparable 5-year OS (90%) to stage II non-screen-detected CRC (88%) and had a statistically significantly superior RFS compared with stage III non-screen-detected CRC. Based on the International Duration Evaluation of Adjuvant Chemotherapy (IDEA) collaboration (19,29), current adjuvant chemotherapy for stage III CRC is informed by dividing patients into low-risk and high-risk groups (as defined by T4 or N2 disease). Given our data on the survival outcomes of screen-detected patients, including those with T4 or N2 disease, this is another patient subset where 3 months of adjuvant therapy may be sufficient treatment. Independent validation of our findings is necessary, however, before routinely factoring in method of detection into adjuvant therapy decision making.

Our data also have potential relevance when considering the surveillance strategy for distant recurrence. Routine surveillance for early-stage disease is part of major guidelines, but randomized studies have not shown a large impact on survival outcomes. If further studies support our findings, given the very low rates of relapse for stage II screen-detected cancers, this group along with all patients with stage I CRC could possibly be considered for less surveillance, if the goal was to minimize the amount of follow-up required without compromising survival outcomes.

Our study has several limitations. We were unable to calculate the number of non-screen- detected CRC that was diagnosed in patients who chose not to participate in the NBCSP or who participated and had a negative test. In addition, patient-related variables, such as lifestyle factors and health-seeking behaviors, are not measured here, which may be contributing to the favorable survival seen in the screen-detected population.

In summary, using a comprehensive registry dataset, this study captures for the first time how the survival benefit of screen-detected CRC is driven by multiple factors beyond an earlier stage at diagnosis. The trifecta of more favorable patient, tumor, and treatment characteristics ultimately culminates in major differences in RFS, OS, and CRC-specific survival for screen-detected patients, including for comparisons within each stage. Further studies will be needed before future adjuvant chemotherapy decision making and surveillance strategies factor in the method of detection as part of an increasingly complex risk stratification model.

## Funding

None.

## Notes

**Role of the funder:** Not applicable.

**Author disclosures:** The authors have no disclosures.

**Author contributions:** SM—conceptualization, formal analysis, investigation, methodology, visualization, writing-original draft, writing-review a editing. WH—methodology, formal analysis, writing-original draft, writing-review & editing. SA—conceptualization, methodology, supervision. IF—data curation, writing-review & editing. IJ—data curation, writing-review & editing. MC—data curation, writing-review & editing. MS—data curation, writing-review & editing. AJ—writing-review & editing. GG—writing-review & editing. YT—writing-review & editing. ML—writing-review & editing. SK—writing-review & editing. RW—writing-review & editing. JT—writing-review & editing. PG—conceptualization, investigation, methodology, resources, project administration, writing-review & editing, supervision, visualization, writing-original draft, writing-review & editing.

**Prior presentations:** Poster presentation at American Society of Clinical Oncology (ASCO) 2021 Gastrointestinal Cancers Symposium GI, January 15-17, 2021. *J Clin Oncol*. 39(3 suppl):42-42. doi: 10.1200/JCO.2021.39.3_suppl.42.

**Acknowledgements:** Michael Harold, Julie Johns, and BioGrid Australia for data collection and collation.

## Data Availability

The data underlying this article will be shared on reasonable request to the corresponding author.

## Supplementary Material

pkab100_Supplementary_Data
