# Supplementary material for: Biology and Clinical Implications of Fecal Occult Blood Test Screen-Detected Colorectal Cancer
Source: JNCI Cancer Spectr. 2022 Jan 10;6(1):pkab100. doi: 10.1093/jncics/pkab100 (PMC8857921; doi:10.1093/jncics/pkab100)
Supplement: pkab100_Supplementary_Data [file pkab100_supplementary_data.pdf]

SUPPLEMENTARY MATERIALS

Supplementary Table 1. Demographics, patient and tumor characteristics of patients with colorectal cancer either screen-detected through the National Bowel Cancer Screening Program or screen-detected outside of the National Bowel Cancer Screening Program.

Characteristic	Screen-detected CRC through the National Bowel Cancer Screening Program	Screen-detected CRC outside of the National Bowel Cancer Screening Program	*P*
Total, No. (%)	473 (53.8)	406 (46.2)	
Mean age at diagnosis (Min, Max)	62.4 (50.2, 74.8)	65.1 (50.2,75.0)	<0.001[a]
Age at Diagnosis, No. (%)			
50-59 y	167 (35.3)	100 (24.6)	
60-69 y	218 (46.1)	184 (45.3)	
≥70 y	88 (18.6)	122 (30.0)	
Sex, No. (%)			0.63[b]
Female	195 (41.2)	161 (39.7)	
Male	278 (58.8)	245 (60.3)	
IRSAD[c], No. (%)			0.31[b]
1-4	87 (18.4)	75 (18.5)	
5-7	169 (35.7)	129 (31.8)	
8-10	205 (43.3)	198 (48.8)	
ASA[d] Score, No. (%)			0.007 [b]
1-2	373 (78.9)	288 (70.9)	
3-5	100 (21.1)	118 (29.1)	
ECOG PS[e], No. (%)			0.06[b]
0-1	447 (94.5)	367 (90.4)	
≥2	8 (1.7)	15 (3.7)	
Unknown	18 (3.8)	24 (5.9)	-
Primary Tumour Site, No. (%)			0.02[b]
Right Colon	147 (31.1)	162 (39.9)	
Left Colon	194 (41.0)	140 (34.5)	
Rectum	132 (27.9)	104 (25.6)	
Tumour Differentiation, No. (%)			0.46[b]
Well-moderately differentiated	361 (76.3)	296 (72.9)	
Poor-undifferentiated	60 (12.7)	57 (14.0)	

Unknown	52 (11.0)	53 (13.1)	
Mucinous Differentiation, No. (%)			0.40[b]
Yes	76 (16.1)	71 (17.5)	
No	315 (66.6)	252 (62.1)	
Unknown	82 (17.3)	83 (20.4)	-
Lymphovascular Invasion, No. (%)			0.59[b]
Yes	121 (25.6)	97 (23.9)	
No	331 (70.0)	289 (71.2)	
T-Stage, No. (%)			0.23[b]
T0-T1	162 (34.2)	128 (31.5)	
T2	78 (16.5)	82 (20.2)	
T3	166 (35.1)	145 (35.7)	
T4	42 (8.9)	25 (6.2)	
Tx (Unknown)	25 (5.3)	26 (6.4)	-
N-Stage, No. (%)			0.2[b]
N0	278 (58.8)	262 (64.5)	
N1	107 (22.6)	82 (20.2)	
N2	46 (9.7)	30 (7.4)	
Nx (Unknown)	42 (8.9)	32 (7.9)	-
AJCC[f] Stage, No. (%)			0.37[b]
I	182 (38.5)	164 (40.4)	
II	100 (21.1)	93 (22.9)	
III	134 (28.3)	98 (24.1)	
IV	26 (5.5)	30 (7.4)	

[a]Unpaired t-test with Welch's correction with two-sided *P* value.

[b]Chi-squared tests were used for categorical data, unless counts were below 10, in which case Fisher's exact test was used. *P* values were two-sided. Patients with missing data for that category were excluded from analysis.

[c]IRSAD: Index of Relative Socio-economic Advantage and Disadvantage.

[d]ASA: American Society of Anesthesiologists (ASA) physical status classification.

[e]ECOG PS: Eastern Cooperative Oncology Group Performance Status.

[f]AJCC: American Joint Committee on Cancer. Note that pathologic staging and detailed histopathologic examination of the primary tumour and locoregional nodes does not routinely occur in de novo Stage IV disease.

Supplementary Figure 1. Relapse free survival across Stage I - III colorectal cancers at diagnosis by method of detection.

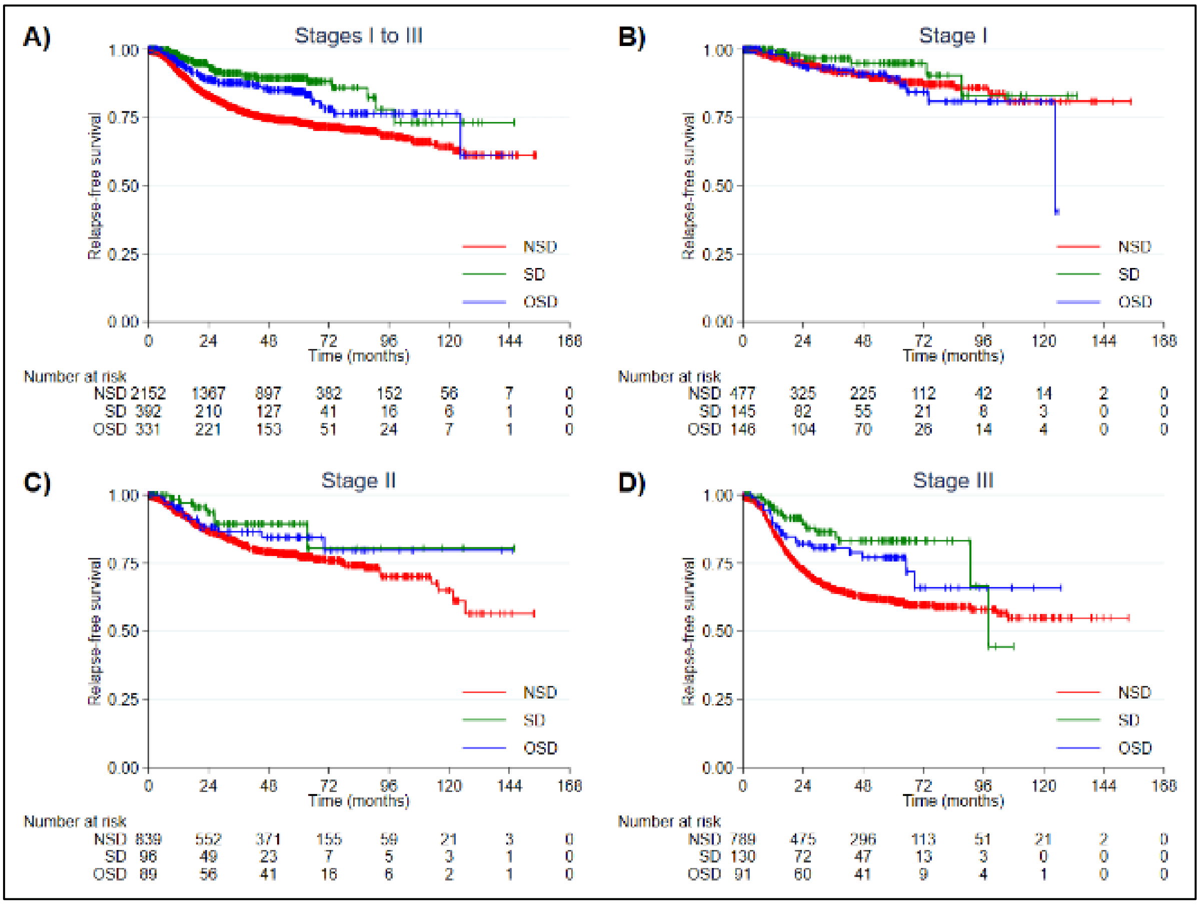

A) Relapse free survival (RFS) for patients with Stage I-III colorectal cancers screen detected within the National Bowel Cancer Screening Program (SD), screen detected outside of the National Bowel Cancer Screening Program (OSD) or non-screen detected (NSD). B) RFS for Stage I. C) RFS for Stage II. D) RFS for Stage III.

Supplementary Figure 2. Overall survival across Stage I - IV colorectal cancers at diagnosis by method of detection.

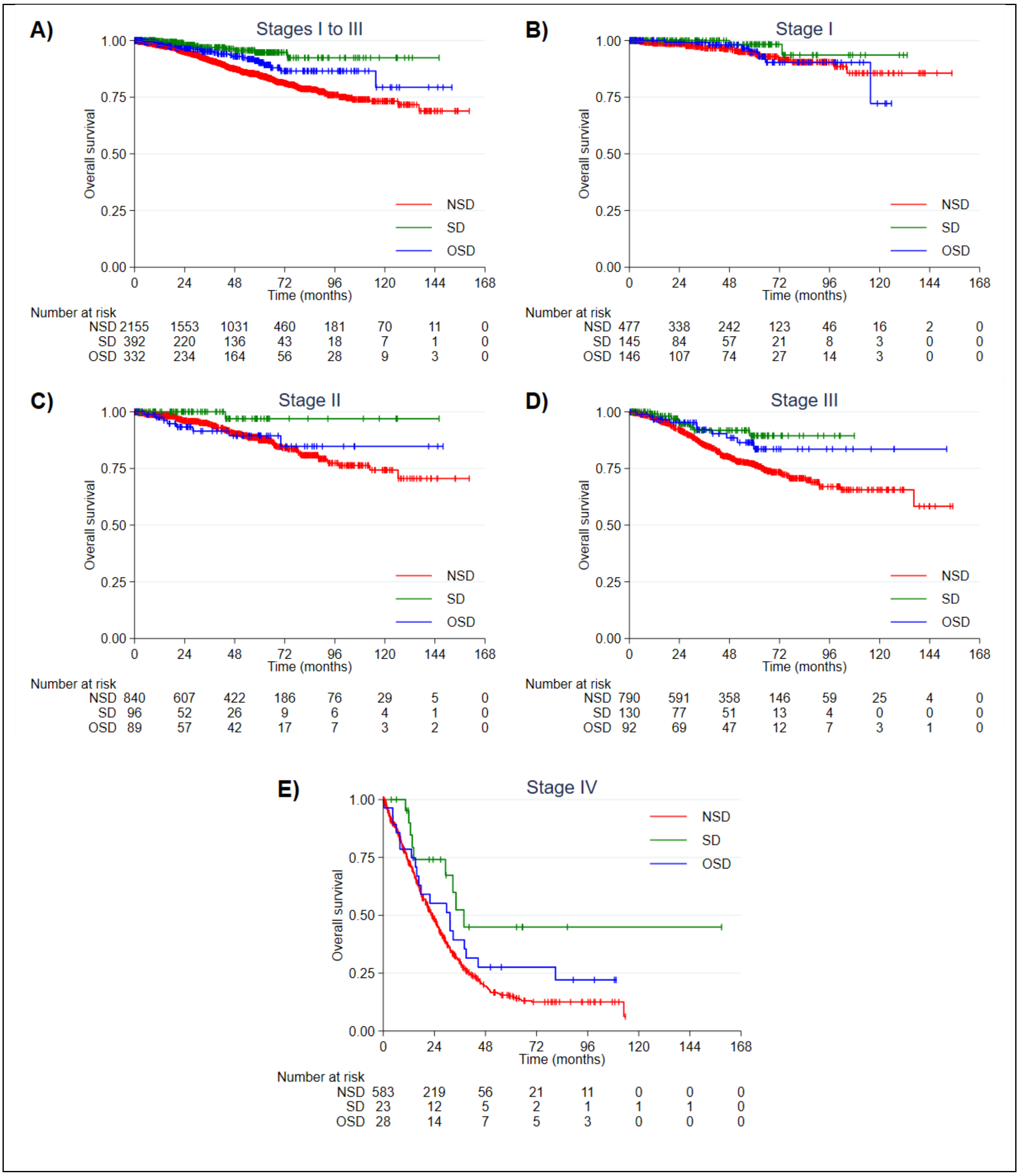

A) Overall survival (OS) for Stage I-III colorectal cancers screen detected within the National Bowel Cancer Screening Program (SD), screen detected outside of the National Bowel Cancer Screening Program (OSD) or non-screen detected (NSD). B) 5-yr OS for Stage I. C) OS for Stage II. D) OS for Stage III. E) OS for Stage IV.
